# Relationship Between Multi-Teaching Styles and Didactics Effectiveness on Rugby Instructors and Minirugby Players

**DOI:** 10.3390/children11111319

**Published:** 2024-10-30

**Authors:** Marta Rigon, Gabriele Signorini, Raffaele Scurati, Athos Trecroci, Dario Colella, Damiano Formenti, Giampiero Merati, Domenico Cherubini, Pietro Luigi Invernizzi

**Affiliations:** 1 Sport Faculty, San Antonio Catholic University of Murcia, 30107 Murcia, Spain; mrigon@alu.ucam.edu (M.R.); dcherubini@ucam.edu (D.C.); 2 Department of Biomedical Sciences for Health, University of Milan, 20129 Milan, Italy; raffaele.scurati@unimi.it (R.S.); athos.trecroci@unimi.it (A.T.); pietro.invernizzi1@unimi.it (P.L.I.); 3 Department of Biological and Environmental Sciences and Technologies, University of Salento, 73100 Lecce, Italy; dario.colella@unisalento.it; 4 Department of Biotechnological and Life Sciences, University of Insubria, 21100 Varese, Italy; damiano.formenti@uninsubria.it (D.F.); giampiero.merati@uninsubria.it (G.M.)

**Keywords:** teaching styles, sport education, competent instructor, didactical success, instructor’s empathy, systems thinking

## Abstract

Background/Objectives: The concept of educational systems thinking shows the rugby educational system as a network of multiple interactive and interconnected elements. However, the frameworks presented in the literature for developing sports practice through an educational vision and multisport engagement do not always have direct transferability to instructors’ training courses. The study aims to evaluate the correlation between rugby instructors’ methodological and didactical competencies and compare them with children’s motor competence and psychological aspects. Methods: Two hundred twenty children (9.6 ± 1.1 years) and 50 instructors (39.0 ± 13.6 years) were enrolled. Instructors’ competencies were investigated during the internship through evaluation sheets, questionnaires, and video analysis. Children’s motor competence and psychological aspects were investigated through motor tests, questionnaires, and video analysis. Results: The correlation between methodological competencies and didactical-communicative competencies showed a positive significance, especially regarding production styles. Motor competence and play skills in children showed poor results. The group of instructors with more methodological competencies (a greater number of teaching styles used) showed a strong correlation between motor competence and psychological aspects. Conclusions: For successful teaching, knowledge of specific technical skills is not enough, and it is important to consider all the factors (in particular, the teaching-communicative and methodological skills) that contribute most to instructors’ skills.

## 1. Introduction

The concept of rugby’s educational system can be defined as a complex dynamic system that includes, from a systems thinking perspective, multiple interactive, interdependent, and interconnected elements [1,2,3]. The dynamic concept, in particular, refers to a current behavior understood in terms of deviations from past behavior that, concerning current regulations [4], envisions a view of sports not only in performance terms but primarily in educational terms [5]. From this perspective, verifying the formative trajectories that make the different professional skills of the instructors interact with the motor and psychological evolution of the children attending the minirugby courses allows us to clarify and define the values and meanings that the Italian Rugby Federation attributes to youth sports practice. This “educational diagnosis” of the teaching reality, based on “Systems Thinking”, represents a contextual mode of analysis. It allows us to adopt targeted actions and to improve and possibly direct and modify non-functional contextual behaviors that interact and can negatively affect the educational concepts that must characterize children’s rugby training [6].

In this way, several frameworks have been concerned with identifying references based on solid pedagogical models for the development of a sports practice aimed not only at performance but also at sports education [7,8]. In an educative vision of sports practice, these pedagogical models must also be applied to children’s sports instructors, as they should extend the sports model to an educational vision that transcends the reductive design of specific interventions with exclusive and limited specialized competitive aims [7,8,9,10,11]. The planning, design, and implementation of model-integrated sports education and initiation pathways [12,13] are characterized by primarily progressive and sequential stages for the education of the body and through the body. These models are intended to guide the development of programs to maintain a positive attitude about sports throughout life. More specifically, they envisage, at the youth level, the construction of physical-motor fundamentals (physical literacy) steps, which are based on a comprehensive multilateral, multi-sport approach [14]. This varied activity makes it possible to add up the training values of different practices while reducing the adverse effects that unilateral sports practice can have [14], also thanks to the attainment of a wider variety of motor skills [15]. However, these models do not always find complete didactic transferability in children’s sports instructor courses, nor has their practical impact on young practitioners always been studied in terms of educational outcomes [10]. Indeed, most federal sports training courses excessively focus on technical elements and performance (ego-oriented [16,17]) that can lead to feelings of inadequacy, excessive competition, and rejection of the activity in their future young athletes, as evidenced by many authors and by an international systematic review [18,19,20]. In the Italian context, rugby instructors’ training courses are proposed by formators of the rugby federation and are differentiated for technical competence of different ages (Level 1: children, Level 2: adolescents, Level 3: adults) and competitive levels (categories of level 3: C, B, A, excellence; categories of level 4: international rugby championship, national team). Future instructors can choose the formation course that better addresses their certification needs. Nevertheless, the courses attribute the qualification of instructor based primarily on “what to teach” and on knowledge and skills (sometimes only theoretical) to be acquired in planning, mastering, and often evaluating purely technical content [21]. This underscores the need for a more balanced approach in sports instructor’s training, focusing on “how to teach” (methodological competence based on teaching styles) and on instructors’ empathic, emotional, and communication skills (didactical and personal competence based on management capacity and psychological constructs), which are indispensable for positively involving pupils [22]. In particular, a task orientation based on pupil-centered teaching styles seems to positively generate a motivational climate [16,23]. The teaching styles are all the various methodologies that teachers and children’s sports instructors can use to efficiently and effectively achieve the set objective [24]. They include various elements such as the teaching strategies used, the different modalities to propose specific experiences, the methods of communication with the children (for example, need-supportive communication), and the instructor’s attitude towards the learners and teaching (instructors’ personal competence; [25,26]). Different teaching styles, especially in sports practice, allow the development of specific skills linked to the psychomotor, emotional, and self-awareness spheres [27,28]. The continuous variation and the wide use of a spectrum of possible teaching styles during sports practice (multi-teaching style approach [29]) can help to better suit the sports didactics to the specific context and the children’s needs. In a new and unconventional way, the multi-teaching style approach can be considered a non-linear [30] use of pedagogical-didactic methods, as it can be continuously modified and adapted to context and situations, and it should be encouraged to solicit multiple and different aspects of children’s identities. Moreover, using different teaching methodologies could also increase instructors’ awareness of his/her educative effectiveness [31]. Conversely, the prevalence of a single teaching style more frequently interpreted as a direct, prescriptive, or command style can be considered a linear and limited approach [29,32]. The multi-teaching style primarily relies on intrinsic motivation and enjoyment in children. This methodological model encourages greater decision-making-oriented involvement of child learners by promoting new stimuli that facilitate the development of motor skills and psychological areas [29]. 

**Figure 1:**
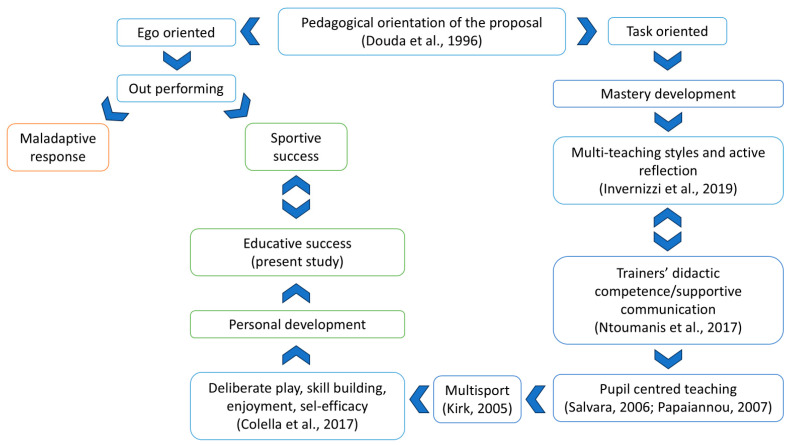
A vision of the youth sports practice aimed at educational and sporting success [14,16,23,26,27,29,35].

In the teaching and sports education processes as well, the combination, variation, and interaction of teaching styles with reflective practices determine different ways of processing information and responses by learners, allowing for different, personalized learning modes [24,29,33]. Hence, the sports instructor focuses on the teaching activity to carry out and thus on how to teach and why. The instructor’s ability to use various teaching styles (methodological competence), didactics competence, and reflection can promote in the child an approach to sports practice that will lead to reaching a healthy and active lifestyle; increased self-awareness and self-body scheme (self-efficacy); greater involvement in sports practice (enjoyment); and maturation of new experiences and motor skills that can also be used in specific sports practice (motor competence) [34]. Figure 1 summarizes the educational vision of sports that underlies this project.

In a sports education based on rugby practice, fundamental educative values such as respect for teammates and opponents, collaboration between team members, and resilience while facing difficulties are promoted. These educative values contribute to sports education beyond technical competencies, considering social and emotional aspects essential for success (individual and collective) and for developing a solid and adaptable character [36]. The teaching duality of sport and educative success in this discipline requires training to develop an adequate practice finalized on objectives that can focus on both dimensions (technical and socio-emotional). Hence, the instructors’ didactical/methodological capacities and the training sessions design become essential when creating a specific environment to improve life and sports competence [37]. Scientific literature considers approaches such as teaching games for understanding and tactical game approaches particularly suitable for this sport. Moreover, the child-centered approach (production teaching styles) is considered adequate for rugby’s sports practice, as it is based on a teaching-learning process conceived on individual characteristics, needs, and motivations [38,39]. Nevertheless, to the best of our knowledge, no studies have specifically addressed the teaching styles used by rugby instructors.

### Aims

The present research analyzed rugby instructors’ didactical (their communicative and organizational abilities and group management) and methodological competencies (the teaching styles they used), investigating the relationships between them. The relationship between motor coordination and psychological aspects (self-perception and enjoyment) in the children trained with and without a multi-teaching methodology by the instructors participating in this study was further analyzed. Finally, instructors’ personal competencies, such as empathy and self-control, and their correlations with the psychological areas (enjoyment and self-efficacy) of children were investigated. 

## 2. Materials and Methods

### 2.1. Sample

Before planning and offering a course for instructors of youth sports, researchers by the University of Milan wanted to explore the context of sports clubs where courses would be applied (which is the core of the present research). Hence, 50 Italian rugby instructors from the youth sector of the local area (two Italian rugby clubs of the Lombardy region) and their respective trained children were recruited. The statistical power resulting from this instructor sample was 0.81. The statistical power was calculated with the G*Power program using an effect size estimate (Cohen’s d) of 0.5. For this calculation, the student *t*-test for unpaired data was chosen. The same calculation was used to estimate the number of children required. An amount of 210 pupils allowed a statistical power of 95%; hence, a sample of 220 children was enrolled. Anthropometric data of children and instructors’ samples are displayed in Table 1.

### 2.2. Instructors’ Background

The rugby instructors participating in the study were all certified by the Italian Rugby Federation (FIR) as “level 1 (children)” instructors. This certification allows instructors to train children from 5 (under 6) to 12 (under 12) years old. Approximately 70% of the recruited instructors had experience as rugby players, 25% came from other sports, and 5% had no sport experience. None of the participants came from a specific sport science university course.

### 2.3. Protocol

Instructors’ behavior (didactic competence) was defined by a list of descriptors used to promote motor tasks and the need for supportive communication. Didactics competencies were assessed during usual rugby training, at the beginning of the sport season, using a modified version of the “Internship Evaluation Sheet in Physical Education and Sports” (IESPES; Table 2; [40,41,42,43]).

Methodological competencies were investigated through the Teaching Styles Questionnaire (TSQ) [44,45], a self-reported questionnaire about the teaching styles used by the instructors.

IFITS and SOFITS systems were employed to verify the effectiveness of the instructors’ use of different teaching styles, the physical activity level, the children’s physical activity level, and the instructor’s interaction capacity during regular rugby training at the beginning of the sports season [46,47].

Instructors’ personal competence was investigated through the Empathy Scale for Teachers (EST) [48] and the Self-Control Questionnaire (SCQ) [49].

In children, for the evaluation of motor competence, we utilized the Körperkoordinationtest für Kinder (KTK) to assess coordination skills [50] and the Game Performance Assessment Instrument (GPAI) to assess game comprehension and sport-specific skills execution [51]. These practical and effective tools were complemented by three questionnaires to investigate self-perception [52], enjoyment [53], and the type of physical activity performed [54].

### 2.4. Measures

#### 2.4.1. Instructor’s Group

1. The Teaching Styles Questionnaire (TSQ; [44,45]) is designed to examine teachers’ teaching style methods, and it has been used to evaluate sports instructors [24]. The TSQ is based on the spectrum of teaching styles classified by [55]. The questionnaire presents for each teaching style a five-point Likert scale score. Points near to 5 express a frequent use of the teaching styles, while points near to 1 mean that it is not used.
2. The Internship Evaluation Sheet in Physical Education and Sports (IESPES; [40,42]) is a tool designed to evaluate the instructors’ didactic competence. A score from zero to five points was attributed to communication capacity (verbal communication, voice, paralanguage, non-verbal communication), didactics organization (specific didactics, elements of organizational references), and capacity to motivate and to engage the pupils (psychological references, personal competence).
3. The Empathy Scale for Teachers [48] is a self-evaluation questionnaire developed to measure educators’ empathy capacity in educational contexts. The questionnaire is composed of 19 questions with answers on a four-point Likert scale. The questionnaire reports an empathy final total score and three sub-scores related to cognitive, positive affective, and negative affective empathy.
4. The Self-Control Questionnaire [49] is an evaluation tool for the measure of self-control. It is composed of items rated on a five-point scale, starting from 1 (“not at all like me”) to 5, (“very much like me”). The questionnaire, composed of 36 items, reports a final self-control total score and eight subscale scores (impulse control, goal setting, distraction control, self-monitoring, task initiation, emotion regulation, automaticity, and decision-making).
5. Video analysis IFITS [47] and SOFIT [46]. Two lessons at the beginning of the sport season were video recorded, and the IFITS and SOFIT analysis systems were used to verify the instructors’ teaching styles, the children’s levels of physical commitment during the lessons, and the interaction capacity of instructors with them. The IFITS instrument gives the % of time spent on each instructor’s teaching style. The SOFIT instrument gives the % of time spent in sedentary behaviors (lay, sit, stand) or moderate to vigorous physical activity (walk or vigorous activity), the % of time spent for each lesson content (skills, game, fitness, management, knowledge, or other contents) and the % of time spent by the instructor in motivating children to activity (motivation focused on the inside lesson topic or outside lesson topic or not motivating).

#### 2.4.2. Children’s Group

1. Körperkoordinationtest für Kinder (KTK, [50]) is a coordinative test for children from 8 to 14 years old. It includes four tests (walking back, jumping sideways, moving sideways, and hopping for height). The result of each test is normalized, converted, and summed into a total motor quotient score (MQ) using a dedicated nomogram. The MQ indicates the children’s level of body mastery that can be allocated into six ranges: disturbed (MQ = 50–70), noticeable (MQ = 71–85), average (MQ = 86–115), good (MQ = 116–130), and excellent (MQ = 131–145).
2. The Physical Activity Questionnaire for Older Children (PAQ-C; [54]) was employed to determine the level of physical activity in the last seven days. Low scores (from 1 to 2.33) correspond to a low level of physical activity; medium scores are related to a moderate level (from 2.34 to 3.66) of physical activity, while high scores (from 3.67 to 5.00) imply a high level of physical activity.
3. The Game Performance Assessment Instrument (GPAI; [51]) is a validated tool that assesses children’s ability to perform during sports games. The instrument evaluates the appropriate decisions or tactical executions, as well as the efficiency of skills performed during the game. In the present analysis, 15 min of matches during rugby training were evaluated. Due to the high number of children, the present study focused the analysis only on the offensive phases and considered the scores performed by the overall team without analyzing single players’ outcomes. The game variables considered were decision-making, skill execution, support, and game performance.
4. The Physical Activity Enjoyment Scale (PACES; [56]) is a 16-item questionnaire that uses a five-point Likert scale (1: completely disagree, 2: disagree, 3: uncertain, 4: agree, 5: fully agree). The questionnaire gives a final total score ranging from 16 to 80; higher values are related to higher enjoyment of the physical activity proposed.
5. The Physical Self-Efficacy Scale for children (PSES; [52]) is a test that evaluates the self-perception of one’s physical efficiency in motor skills, considered a primary motivational factor for voluntary participation in any physical activity and sport. It is composed of six questions and comprises a four-point Likert scale. The questionnaire gives a final total score ranging from 6 to 24; higher values are related to a more positive perception of the self.

This study was approved by the ethical committee of the University of Milan on 13 February 2024 with opinion number 14/24.

### 2.5. Reliability of Internship Evaluation Sheet and Video Analysis

To evaluate the intra- and inter-rater reliability of IESPES, SOFIT, and IFITS, a video of 10 instructors’ internships and the children’s training (six trainings) was recorded. Three different operators who were adequately trained to use the internship evaluation sheet analyzed the video. Every operator repeated the evaluation after two weeks. The video sequence was randomly assigned. 

The GPAI intra- and inter-rater reliability process followed the same procedure but was based on videos of two different matches. 

### 2.6. Statistical Analysis

The normal data distribution has been verified using the Shapiro-Wilk test. 

The intra-rater and inter-rater reliability of IESPES was assessed using the intraclass correlation coefficient (ICC). A reliability value above 0.70 was considered sufficient for the evaluation. 

For the GPAI, IFITS, and SOFIT evaluation reliability, the following formula was used to assess the percentage of agreement between and within raters [57,58]:

% agreement = n° agreement/(n° agreement + n° disagreement) × 100

An agreement rate of over 80% was deemed necessary for the test to be considered reliable. 

The differences in IESPES, self-reported teaching styles, IFITS, SOFIT, KTK, and GPAI results were investigated using the two-way ANOVA or the respective non-parametric Friedman test.

The Spearman correlation was performed to investigate the relationship between instructors’ teaching styles and instructors’ didactic competence (IESPES). Moreover, to assess relationships between instructors’ characteristics and children’s outcomes, two groups of instructors, a multi-teaching group (MG), and a non-multi-teaching group (NMG) were established considering the results of the self-reported questionnaire (TSQ). Instructors who used many teaching styles were enrolled in the MG, and those who used only one or two teaching styles were enrolled in the NMG. In this division, we used the results of the teaching styles questionnaire and not the results of IFITS because the former concerned the entire experience of the instructor, while the latter referred to the observational analysis of only two lessons. 

Spearman’s correlation was applied to relate (i) self-reported teaching styles and IESPES outcomes; (ii) KTK normalized score and enjoyment in multi-teaching children; (iii) KTK normalized score and enjoyment in non-multi-teaching children; (iv) KTK normalized score and self-efficacy in multi-teaching children; and (v) KTK normalized score and self-efficacy in non-multi-teaching children. 

Finally, to assess relationships between children’s psychological aspects (enjoyment and self-efficacy) and instructors’ personal competencies (empathy and self-control), no instructor group division was performed, as we used the percentages of the questionnaires’ results. Hence, the correlations were assessed using Spearman’s correlation. 

For all mentioned analyses, the level of significance was set at 0.05.

A descriptive statistic was used for PACES, PSES, and PAQ-C general outcomes.

## 3. Results

### 3.1. Reliability Results

The IESPES obtained values of intra-rater ICC higher than 0.852 and inter-rater ICC higher than 0.886. GPAI, IFITS, and SOFIT evaluations obtained values of agreement higher than 82% for intra- and inter-rater reliability.

### 3.2. Instructors’ Descriptive Results

#### 3.2.1. IESPES Results

**Figure 2:**
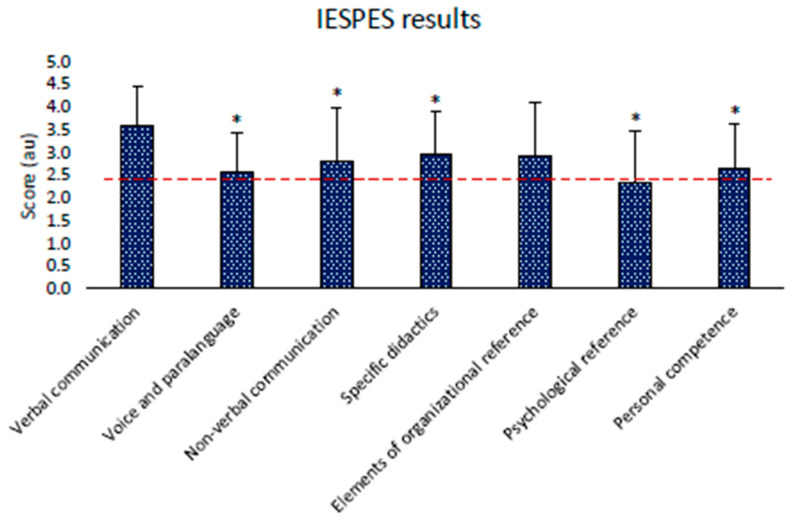
IESPES results. The red dashed line represents the minimum teaching competence score. Difference with “verbal communication”: * = *p* < 0.05.

The ANOVA test revealed significant differences in IESPES results (*p* < 0.001, ηp2 = 0.13). Post hoc analysis revealed that verbal communication was the higher value obtained by rugby instructors (*p* < 0.05). Only the organizational elements item did not significantly differ from verbal communication in the post hoc analysis (*p* = 0.09). The results are displayed in Figure 2.

#### 3.2.2. Self-Reported Teaching Styles Questionnaire’s Results

**Figure 3:**
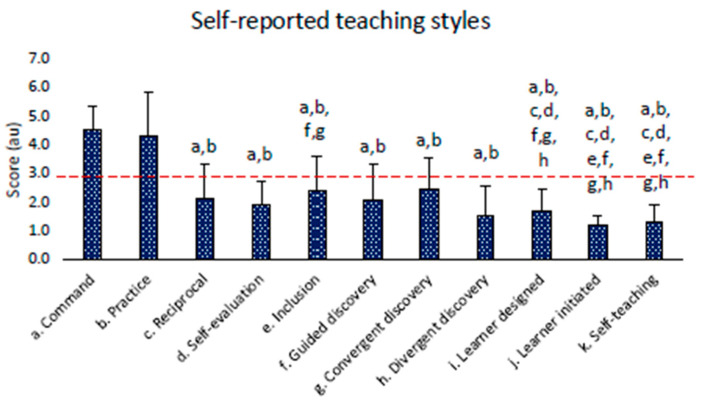
Results of the self-reported teaching styles questionnaire. The red dashed line represents the value referred to as the “here and there” descriptor and was interpreted as the minimum arbitrary value (set at 60% of the total score) to consider a teaching style sufficiently used during training. Letters indicate a significant difference with the respective teaching style (*p* < 0.05).

The Friedman test for the self-reported teaching styles resulted in significance (*p* < 0.001). Post hoc analysis revealed significantly higher values for the command and practice (a and b) teaching styles (*p* < 0.05) and lower values for all other teaching styles. All post hoc analysis results are displayed in Figure 3.

#### 3.2.3. Teaching Styles’ Video Analysis Results (IFITS)

**Figure 4:**
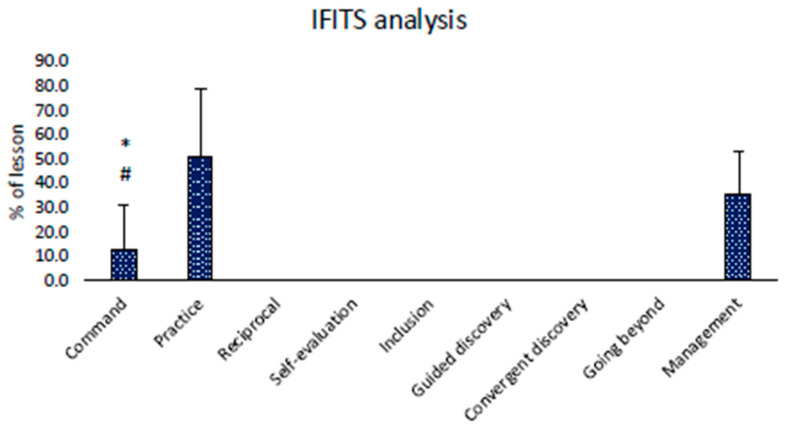
Results of IFITS analysis. Different than practice: * = *p* < 0.05. Different than management: # = *p* < 0.05.

The Friedman test resulted in significance for IFITS analysis (*p* < 0.001). The post hoc analysis revealed that command style and practice are the only styles used, confirming the prevalence of these methodologies. Between the two, in the lessons analyzed, the command style was the teaching style less frequently used (13.2 ± 18.2 % of lesson, *p* = 0.004), while most of the time was spent with the practice teaching style (51.1 ± 28.4 % of lesson) and management activities (35.7 ± 17.7 % of lesson). Results are displayed in Figure 4.

#### 3.2.4. Physical Activity’s Video Analysis Results

**Figure 5:**
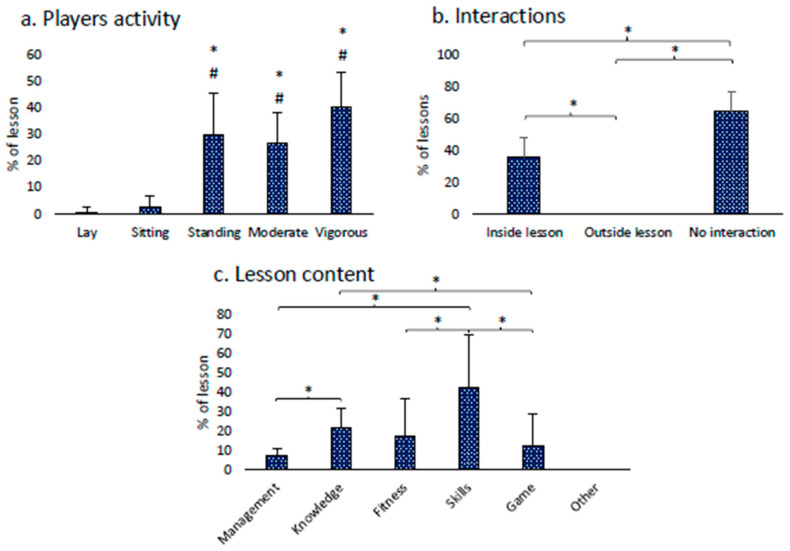
Results of SOFIT players activity (**a**), instructors interactions (**b**), and lesson content (**c**). Panel (**a**): different than lay: * = *p* < 0.05; different than sitting: # = *p* < 0.05. Panel (**b**): significant difference: * = *p* < 0.05. Panel (**c**): significant difference: * = *p* < 0.05.

Friedman’s analysis of players’ activity showed significant differences (*p* < 0.001). The post hoc analysis revealed that the main part of the lessons was spent in standing (30.1 ± 15.4% of the lesson), moderate (26.8 ± 11.6% of the lesson), and vigorous (40.1 ± 13.2% of the lesson) physical activity. Concerning instructors’ interaction with children, the Friedman analysis showed that most instructors did not interact with children during lessons, few of them interacted by focusing on inside lesson topics, and none of them interacted by focusing on outside lesson topics. Finally, the main content of rugby lessons was directed toward improving specific rugby skills. Figure 5 shows all significant differences. 

#### 3.2.5. Empathy Scale for Teachers (EST) Results

**Figure 6:**
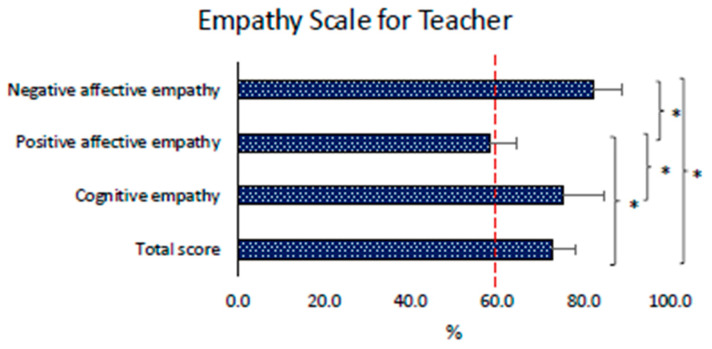
Instructors’ empathy descriptive results. The red dashed line represents the cut-off line of low (<60%) or high (>60%) empathy. * = significant difference (*p* < 0.05).

Regarding instructors’ empathy, the positive affective empathy result was the lower value (*p* < 0.05) and the one lower than 60%, while the higher values reported were the negative affective empathy and the cognitive empathy. The results of the Empathy Scale for Teachers are displayed in Figure 6.

#### 3.2.6. Self-Control Scale Results

**Figure 7:**
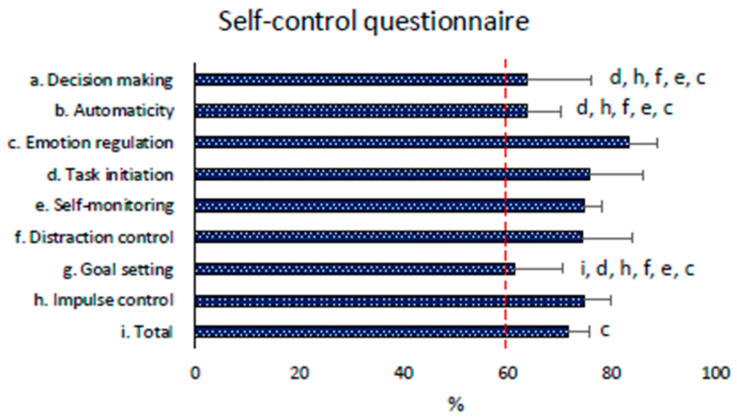
Instructors’ self-control descriptive results. The red dashed line represents the cut-off line of low (<60%) or high (>60%) self-control. Letters indicate significant difference with the respective self-control scale (*p* < 0.05).

The Self-Control Questionnaire revealed that the instructors’ sample had high values of emotion regulation, task initiation, self-monitoring, distraction, and impulse control. Conversely, the sample reported low values of decision-making, automaticity, and goal-setting (all close to 60% of the maximal reachable score). The statistical results of Friedman’s comparison are reported in Figure 7.

### 3.3. Children’s Descriptive Results

#### 3.3.1. Körperkoordinationtest für Kinder Results (KTK)

**Figure 8:**
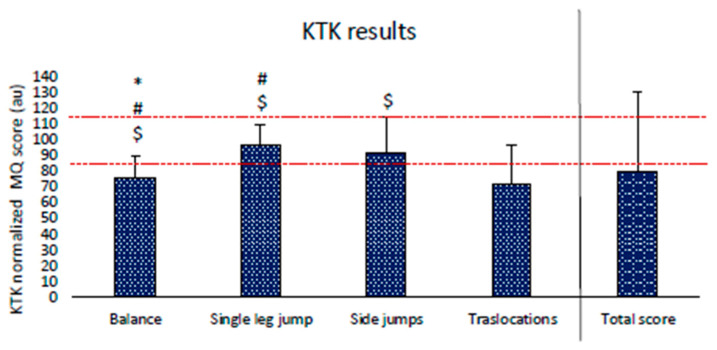
Results of KTK analysis. Significantly different than translocations: $ = *p* < 0.05; significantly different than side jumps: # = *p* < 0.05; significantly different than single leg jump: * = *p* < 0.05. Red dashed lines represent the coordination normality range between 85 and 115 au. MQ = motor quotient.

The two-way ANOVA revealed a significant difference between KTK sub-tests (*p* < 0.001). The post hoc analysis revealed that the higher scores were performed in single leg jump (96.3 ± 12.9 au) and side jumps (92.1 ± 23.1 au), which reached the normal score range (between 85 and 115 au). Other results are displayed in Figure 8.

#### 3.3.2. Game Performance Assessment Instrument Results (GPAI)

**Figure 9:**
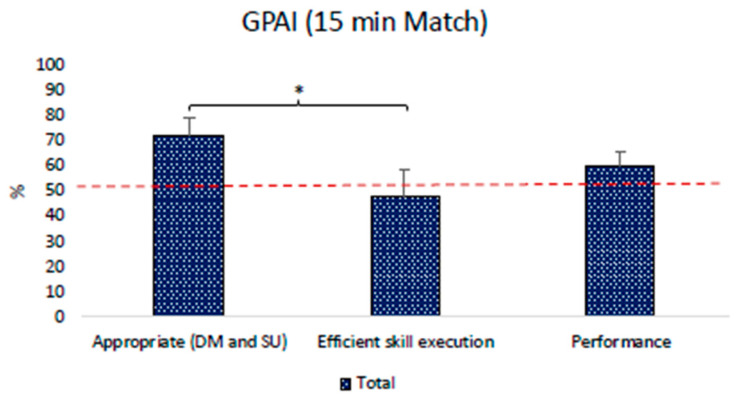
GPAI results. The red dashed line represents 50% of the total decision and execution performed. * = *p* < 0.05. Appropriate (DM and SU) = % of appropriate choices on the total choices (summing decision-making choices and teammate support) determined by the team; efficient skill execution = % of efficient skills execution on the total of the skills executed during the video recording; performance = % of success related to the efficient execution of the appropriate choice made by children [59].

The Friedman analysis revealed that the children made higher percentages of appropriate decisions (71.5 ± 7.7%) compared with efficient skill execution, which resulted in low percentages (47.8 ± 10.5%). No differences were detected with performance level (59.7 ± 5.8%). Results are displayed in Figure 9.

#### 3.3.3. Questionnaires Descriptive Results

Table 3 displays the results of the questionnaire and KTK reached by children and the % of maximal reachable points. 

### 3.4. Correlation Results

#### 3.4.1. Correlation Between Teaching Styles and IESPES Results

**Figure 10:**
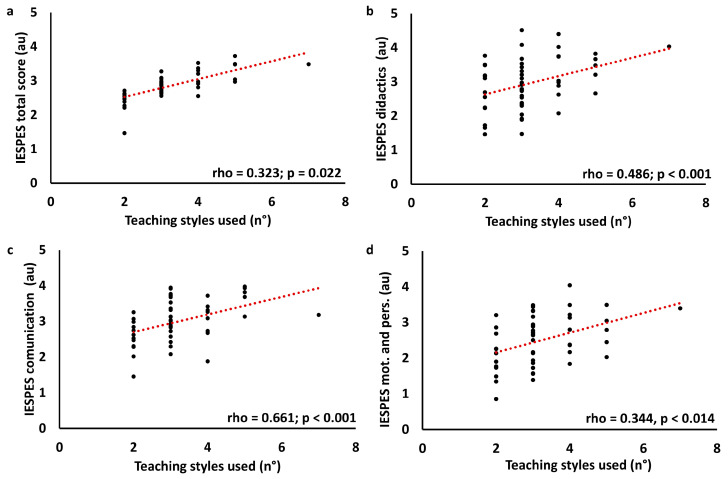
Correlation graph between IESPES scores and the number of teaching styles prevalently used. IESPES scores: total score (**a**), didactics (**b**), communication (**c**), motivation and personal competence (**d**).

**Figure 11:**
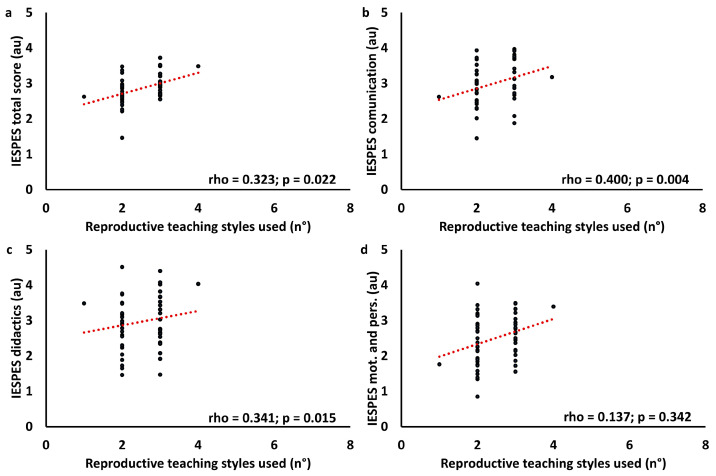
Correlation graph between IESPES scores and the number of reproductive teaching styles prevalently used. IESPES scores: total score (**a**), didactics (**b**), communication (**c**), motivation, and personal competence (**d**).

**Figure 12:**
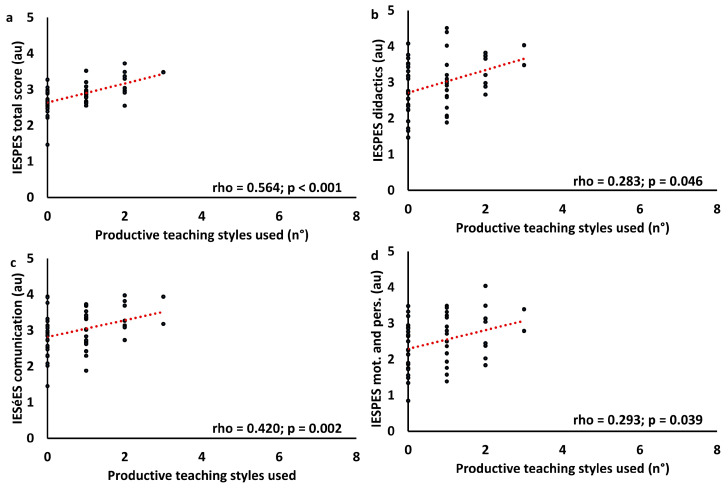
Correlation graph between IESPES scores and the number of productive teaching styles prevalently used. IESPES scores: total score (**a**), didactics (**b**), communication (**c**), and motivation and personal competence (**d**).

Correlation between teaching styles questionnaire results and IESPES results has been performed. For this analysis, the items “verbal communication”, “voice”, and “non-verbal communication” were summed in the “communication” category. In the same way, “didactics” and “organizational elements” were summed in the “didactics” category, while “psychological elements” and “personal competence” were summed into the “motivation and personal competence” category. Moreover, teaching styles questionnaire results were summarized in “multi teaching” (number of teaching styles evaluated as four and five, respectively “very often” and “most of the time”), reproduction (number of reproductive teaching styles evaluated as four or five), and production (number of productive teaching styles evaluated as four or five). Data were analyzed using Spearman’s correlation. Results are summarized in Table 4. Primary correlations are displayed in Figure 10, Figure 11 and Figure 12.

#### 3.4.2. Correlation Results Between Motor Competence, Self-Efficacy, and Enjoyment in Children with Multi-Teaching and Non-Multi-Teaching Instructors

**Figure 13:**
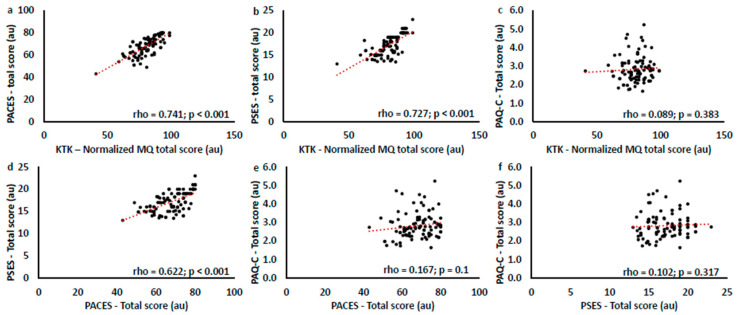
Correlation graphs between KTK and PACES (**a**), KTK and PSES (**b**), KTK and PAQ-C (**c**), PACES and PSES (**d**), PACES and PAQ-C (**e**), and PSES and PAQ-C (**f**). The sample considered is children with multi-teaching trainers.

**Figure 14:**
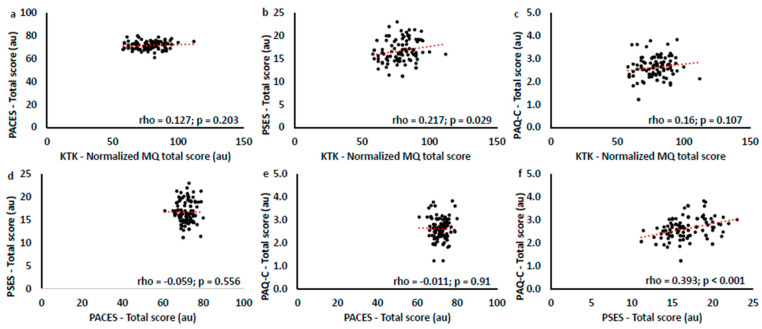
Correlation graphs between KTK and PACES (**a**), KTK and PSES (**b**), KTK and PAQ-C (**c**), PACES and PSES (**d**), PACES and PAQ-C (**e**), and PSES and PAQ-C (**f**). The sample considered is children with non-multi-teaching trainers.

Spearman’s correlations were performed to evaluate the relationships between motor competence and psychological aspects in children with multi-teaching and non-multi-teaching trainers. Results are reported in Table 5 and Table 6 and in Figure 13 and Figure 14.

#### 3.4.3. Correlation Analysis of Enjoyment and Self-Efficacy with Instructors’ Empathy and Self-Control

Spearman’s correlations were performed to evaluate the relationships between children’s psychological aspects (enjoyment and self-efficacy) and instructors’ interpersonal competence (empathy and self-control). The main results are shown in Table 7 and Table 8, while Appendix A (Table A1) displays the complete correlation table.

## 4. Discussion

The present research aimed to evaluate the correlation between the rugby instructors’ methodological competencies and their didactic competence. Moreover, this study aimed to evaluate and compare the effects of the variation in the teaching styles used on motor competence, self-perception, and enjoyment in children who received a multi-teaching style approach and children who did not.

From the results that emerged from this study, considering a mean score of 3 au (60% of maximal score), we can affirm that concerning didactics competencies, the instructors had good verbal communication skills, sufficient organizational skills, and specific didactics skills (exercises’ choices, demonstrations, corrections, etc.). Nevertheless, they reported poor non-verbal communication (facial expressions, posture, gestures, proxemic gestures, etc.), personal competence (charisma, empathy, self-monitoring, etc.), skills in managing voice and paralanguages (volume, timbre, modulation, etc.), and psychological references (interest arouse, emulative level, reinforcements, etc.). Considering the statistical analysis, organizational skills were the only describer that did not differ from verbal communication. All the other describers indicated the need for intervention oriented to their improvement. The capacity to positively manage the different didactic competencies represents a key element in understanding how to help young people develop and maintain adequate physical literacy for sports and educational success [60]. Concerning methodological competencies, according to a recent study, instructors revealed, through self-reported teaching styles, a prevalent use of the command and practice styles. In line with our results, Fernandez et al. (2021) reported that federal instructors use these styles more frequently; conversely, specialized PE teachers use a multi-teaching styles approach in sports education [61].

Video analysis (IFITS analysis) showed that instructors exclusively used command and practice styles (mainly used). Furthermore, substantial time was needed to manage the activities, demonstrating poor employment of effective didactics. The instructors’ poor time management capacity seems to contradict the IESPES organizational skills outcomes; nevertheless, the time management represents just one of the five descriptors that compose the evaluation. This should mean that even if rugby instructors are sufficiently able to spatially organize the lesson (considering location of tools, location of learners, safety/prevention/assistance rules, use of conceptual illustrations), they have difficulties in managing time during training. The children’s sports sphere represents the most appropriate sample to experiment with and check various learning approaches that could stimulate active participation and personal development. Nevertheless, the limited instructors’ didactics and methodological skills represent a significant barrier to achieving these objectives [62].

Concerning physical activity involvement evaluated through the SOFIT system, time spent in moderate and vigorous physical activity (MVPA) is greater than time spent sitting or lying, but considerable time is spent staying still. Deepening the analysis, the contents of the proposed activities are much more dedicated to skills learning than games and fitness, similar to cases highlighted by literature [63]. The instructor-children relationship during lessons is poor, and feedback is only related to the on-field practice. No instructor promotes the transferability of the practice of other activities or contexts necessary to promote a multi-sportive education and a more comprehensive promotion of physical activity even outside sport-specific practice [64]. 

The Self-Control Questionnaire and the Empathy Scale for Teachers, which highlight higher levels of emotion regulation, and low results of positive affective empathy can constitute two personal competencies to be considered as elements to be improved to promote educational interaction with pupils [65,66].

Correlation analysis between internship score (didactic competence) and the number of teaching styles used (methodological competence) showed a positive relationship between multi-teaching productive style and the total score reached in total didactic competence, organizational elements, specific didactics, motivation, and personal competence of the instructor. The correlations of the didactic competence with the number of reproductive styles used show the same results, except for the motivation/personal competence. These results agreed with Rivera-Perez (2020), who showed that multi-teaching motor tasks, both with reproduction styles (reciprocal, self-check following specific criteria) and production styles (divergent discovery for small groups), favor the development of soft skills emphasized with relationship dynamics in children and adolescents [28]. Communication skills are more related to positive correlations with didactics’ organizational and managing aspects, motivation/personal competence, and total score, confirming that didactics could be considered a communication science [43].

Considering the results of the analyzed children sample, the outcomes of motor competence in children present under motor normality levels, referring to the motor quotient of the KTK test. Similarly, game performance, determined by GPAI, evidenced results of less than 50% of effective skills executions despite the appropriate choices related to situational needs. The acquisition of adequate coordination could positively affect sport-specific skill learning. Indeed, skill competence could improve game comprehension, reducing failure after a good tactical choice depended on bad skill management [67]. Notably, El Khouri evaluated the effects of two learning units based on command and guided discovery styles on the gymnastics skills of two groups of children [68]. Although learning results were similar in the two groups, the guided discovery styles guarantee better learning retention from the medium to long term than the command styles. Furthermore, Da Silva showed how production styles, weakly used in our study by the rugby instructors, improved the learning of sport-oriented skills in team sports, such as decision-making, appropriate choice selection, and being active and involved in the games [69].

Preliminary results of the children’s questionnaires evidenced high scores related to enjoyment and self-efficacy but insufficient general physical commitment of children concerning the total physical activity done. The correspondence between teaching and learning is determined by the choices and didactical decisions made, the questions the instructor formulates, and the expected responses (related to what task and how to execute). As previously highlighted in SOFIT, the poor interaction of the instructors in promoting further different sports and activities outside of rugby lessons may have influenced these results [63,64]. Referring to the correlation between children’s psychological constructs (physical self-perception and enjoyment) and motor competence in MG and NMG, MG showed a strong correlation with motor competence, enjoyment, and physical self-efficacy, and NMG showed a weak correlation between competence and self-efficacy. Self-perception and enjoyment development are interdependent because they are the outcomes of motor tasks successfully executed and from their previous skills. Understanding how teaching styles vary and are interconnected is crucial for assessing the effects on motor learning and related psychological factors [70,71]. Motor competence is related to the type and variety of teaching styles used for positive living and their relationship with children’s self-perception, which could sustain motor and sports practices [52,53]. This analysis suggests that research perspectives will have to proceed in different and complementary directions: on the one hand, through the sports training of instructors through evidence-based didactics, and on the other, through analyses and studies geared towards enhancing not only motor performance (sporting success) but also the underlying psychological and social factors (educational success) [16,17]. Rugby instructors’ training courses must include significant experience in sports activity methodology and psycho-pedagogical sensitivity for children and young people. 

Instructor’s empathy and self-control are closely related to the children’s positive psychological approach to the lessons [66]. More specifically, in our study, empathy correlates with enjoyment and self-efficacy in children. These results are consistent with the literature, highlighting how this personal competence is an indispensable characteristic of an engaging instructor who does not demotivate children [25]. Regarding self-control, an instructor’s excess of dirigisme and inhibitory behavior could negatively affect the children’s intrinsic motivation and determine relational problems [72]. In particular, an excessive definition of objective can lead to an excessive definition of executive rules with a greater possibility of children’s failure and a lower sense of self-efficacy [73]. Similarly, the teacher with an excess of decision-making takes away decision-making autonomy from the children and, therefore, negatively influences their perception of self-efficacy. Regarding the relationship between self-control and enjoyment, an excess of impulse control and automaticity could reduce teacher creativity and spontaneity in lessons; an excess of task initiation could induce excessive pressure with a misunderstanding of the proposal and minor engagement [74,75,76]. A more frequent use of student-centered production styles and supportive communication could help solve these problems [23,26,77]. This concept agrees with the principle of self-determination theory and need-supportive communication. It highlights how autonomy and the discreet presence of the instructor, who must be present but let children experiment without doing things in their place, are critical elements of an effective sport and formative success with children [26]. Finally, correlation results highlighted a direct relationship between both enjoyment and self-efficacy with self-monitoring (as part of the Self-Control Questionnaire). This means that a more reflective instructor who knows how to think about his/her behavior, proposal, and relationships during a training session can modify, implement, and improve the lesson for the pupils he/she is referring to when required. This way, the instructors’ self-monitoring capacity could lead to a higher enjoyment of the sports practice and a higher sense of efficacy in children [29,78].

### Limitations of the Study

Two main limitations can be acknowledged in the present study. The geographical area of the rugby clubs from which participants were involved in the research was limited to the local area near the researchers’ university in northern Italy. This could represent a relevant issue possibly affecting the generalizability of the results as sensible differences with other local areas surely exist with respect to facilities and/or rugby popularity and diffusion. Moreover, rugby is a sport prevalently practiced in Italy by male players and has few young female rugby players. For this reason, gender comparison has not been possible.

## 5. Conclusions

**Figure 15:**
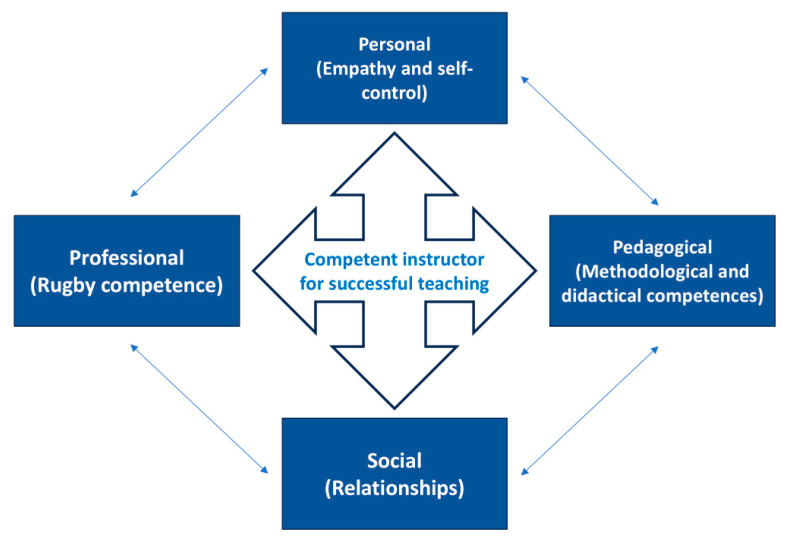
Summary table of the relationship between the emerging learning skills of this study.

In a concept of rugby-oriented system thinking, our study well evidences the relationship between different instructors’ learning skills and educational success (Figure 15). 

The multi-teaching methodological approach and the connection with instructors’ communicative teaching and empathic engagement skills positively influence the relationships between motor competence and the effects on children’s psychological components. This interconnection highlights the importance of orienting instructor training toward the ability to use varied teaching styles, especially production styles, which is a key leverage point for the educational success of their practice [29]. 

A point of resistance that emerges from this analysis is the pupils’ poor general motor competence, evidenced by the KTK, the GPAI, and the SOFIT observation of the instructors’ poor interaction and involvement in promoting multilateral and multi-sportive motor practice in children, also carried out in the lessons [64]. Loop research between instructor/children variables to determine the effects of reciprocal interactions (circular loop based on “causal relationship chains” of direct and opposite return directions) supports a training approach based on instructors’ methodological learning [79]. 

Instructors’ use of different teaching styles and multisport and multilateral practice may constitute the most critical variations in an approach that must always be oriented more toward educational success than only toward young people’s sports performance.

### 5.1. Practical Application

The present research established that training rugby instructors utilizing a multi-teaching reflective approach and implementing the children’s interaction in promoting multi-sports activity outside lessons [14,29] could effectively contribute to creating the proper educational and methodological competence oriented to support both the sport and the educational success of young rugby players. In addition, empathic and self-monitoring capacities resulted related to children’s psychological well-being, expanding the need for qualified instructors having technical skills to develop didactical and methodological competencies granting positive motor and psychological results. That can favor rugby performance and the development of a child’s physical literacy.

### 5.2. Future Research Development

Studies and research need to be more frequently and systematically integrated into the settings where the teaching process occurs. In addition, studies on the teaching methods implemented in the different disciplines should be expanded and made more common to indirectly acquire knowledge on how children learn sports motor skills. Future studies should focus on the efficacy and transferability of instructors’ courses based on a multi-sports and multi-teaching reflective approach to advance scientific evidence.

## Figures and Tables

**Table 1:** Anthropometric data of the sample.

	Children	Instructors
Sample size (n°)	220	50
Age (years)	9.6 ± 1.1	39.0 ± 13.6
Males (n°)	216	45
Females (n°)	4	5
Weight (Kg)	36.0 ± 7.5	
Height (m)	1.4 ± 0.1	
BMI (kg/m^−2^)	18.6 ± 3.3	
Rugby teaching experience (years)		6.0 ± 5.7

**Table 2:** IESPES items’ descriptors.

Items	Descriptors ^1^
Verbal communication	Comprehension; logic; choice of words of appreciation; choice of words of reproach; use of metaphors/narration.
Voice and paralanguage	Volume; timbre; modulation; expository rhythm; use of silences and pauses.
Non-verbal communication	Facial expression; eye gaze; eye contact; posture; proxemics gestures.
Specific didactics	Choice of exercises; progression; demonstration; correction; adaptation of the proposal.
Elements of organizational references	Location of tools; location in space of learners; safety/prevention/assistance rules; use of conceptual diagrams/charts/illustrations; time management (activity times; question times; organizational times).
Psychological references	Interest aroused; emulation level; expectations/requests; reinforcement; contextualization.
Personal competence	Charisma; accuracy; confidence; empathy; self-monitoring

^1^ Instructors were evaluated on five descriptors for each item [40,41,42,43]. The descriptors were scored as 0 (=inadequate) or 1 (=adequate) point, returning a score ranging from 0 to 5 for each item. The final score was calculated as the mean of all items’ scores.

**Table 3:** Questionnaire results and % of maximal reachable point.

	Total Scores ^a^	Maximal Reachable Points	% of Maximal Reachable Point ^a^
PACES (au)	69.8 ± 6.6	80	87.3 ± 8.2
PSES (au)	17.0 ± 2.4	24	70.6 ± 9.9
PAQ-C (au)	2.7 ± 0.6	5	54.6 ± 12.0

^a^ Mean ± standard deviation.

**Table 4:** Results of correlation analysis between teaching styles and IESPES results.

		Multi-Teaching	Reproduction	Production	Communication	Didactics	MPC ^1^	Total Score
Multi-teaching	rho	1	−0.136	0.195	0.661 **	0.486 **	0.344 *	0.323 *
	*p*-value	-	0.346	0.174	<0.001	<0.001	0.014	0.022
Reproduction	rho	-	1	−0.072	0.400 **	0.341 *	0.137	0.335 *
	*p*-value	-	-	0.618	0.004	0.015	0.342	0.017
Production	rho	-	-	1	0.564 **	0.420 **	0.283 *	0.293 *
	*p*-value	-	-	-	<0.001	0.002	0.046	0.039
Communication	rho	-	-	-	1	0.747 **	0.479 **	0.542 **
	*p*-value	-	-	-	-	<0.001	<0.001	<0.001
Didactics	rho	-	-	-	-	1	0.538 **	0.792 **
	*p*-value	-	-	-	-	-	<0.001	<0.001
MPC ^1^	rho	-	-	-	-	-	1	−0.04
	*p*-value	-	-	-	-	-	-	0.782
Total score	rho	-	-	-	-	-	-	1
	*p*-value	-	-	-	-	-	-	-

^1^ MPC = Motivation and personal competence. * = *p* < 0.05, ** = *p* < 0.001.

**Table 5:** Correlation results between motor competence (KTK, PAQ-C), self-efficacy (PSES), and enjoyment (PACES) in children with multi-teaching trainers.

Children with Multi-Teaching Trainers		Normalized Total MQ	PACES	PSES	PAQ-C
Normalized total MQ	rho	1	0.741 **	0.727 **	0.089
	*p*-value	-	<0.001	<0.001	0.383
PACES	rho	-	1	0.622 **	0.167
	*p*-value	-	-	<0.001	0.1
PSES	rho	-	-	1	0.102
	*p*-value	-	-	-	0.317
PAQ-C	rho	-	-	-	1
	*p*-value	-	-	-	-

** = *p* < 0.001.

**Table 6:** Correlation results between motor competence (KTK, PAQ-C), self-efficacy (PSES), and enjoyment (PACES) in children with non-multi-teaching trainers.

Children with Non–Multi-Teaching Trainers		Normalized Total MQ	PACES	PSES	PAQ-C
Normalized total MQ	rho	1	0.127	0.217 *	0.16
	*p*-value	-	0.203	0.029	0.107
PACES	rho	-	1	−0.059	−0.011
	*p*-value	-	-	0.556	0.91
PSES	rho	-	-	1	0.393 **
	*p*-value	-	-	-	<0.001
PAQ-C	rho	-	-	-	1
	*p*-value	-	-	-	-

* = *p* < 0.05, ** = *p* < 0.001.

**Table 7:** Main results of enjoyment correlation table.

(a)	Empathy												
	Enjoyment		Total %			Cognitive Empathy %			Positive Affective Empathy %			Negative Affective Empathy %	
Enjoyment	1		0.281 **			0.135			0.726 **			0.019	
**(b)**	**Self-Control**												
	**Total**	**IC**		**GS**	**DC**		**SM**	**TI**		**ER**	**AU**		**DM**
Enjoyment	0.158 *	−0.202 **		0.296 **	0.098		0.322 **	−0.298 **		0.105	−0.255 **		0.158 *

Tables show rho values of the correlation of children’s enjoyment vs. instructor’s empathy variables (7a) and children’s enjoyment vs. instructor’s self-control (7b) variables. IC = impulse control, GS = goal setting, DC = distraction control, SM = self-monitoring, TI = task initiation, ER = emotion regulation, AU = automaticity, DM = decision making. * = *p* < 0.05, ** = *p* < 0.01.

**Table 8:** Main results of the self-efficacy correlation table.

(a)	Empathy												
	Enjoyment		Total %			Cognitive Empathy %			Positive Affective Empathy %			Negative Affective Empathy %	
Self-efficacy	1		0.397 **			0.288 **			0.288 **			0.223 **	
**(b)**	**Self-Control**												
	**Total**	**IC**		**GS**	**DC**		**SM**	**TI**		**ER**	**AU**		**DM**
Self-efficacy	−0.107	−0.013		−0.325 **	−0.031		0.296 **	0.035		−0.092	0.092		−0.324 **

Tables show rho values of the correlation of children’s self-efficacy vs. instructor’s empathy variables (8a) and children’s self-efficacy vs. instructor’s self-control (8b) variables. IC = impulse control, GS = goal setting, DC = distraction control, SM = self-monitoring, TI = task initiation, ER = emotion regulation, AU = automaticity, DM = decision making. ** = *p* < 0.01.

## Data Availability

The data presented in this study are available on request from the first and second authors due to privacy reasons.

## Appendix A

**Table A1:** Correlation analysis of enjoyment and self-efficacy with instructors’ empathy and self-control.

		PACES	PSES	Empathy Scale for Teachers				Self-Control Scale								
		Enjoyment	Self-Efficacy	Total%	CE%	PAE%	NAE%	Total%	IC%	GS%	DC%	SM%	TI%	ER%	AU%	DM%
PACES	Enjoyment	1	0.303 **	0.281 **	0.135	0.726 **	0.019	0.094	0.158 *	−0.202 **	0.296 **	0.098	0.322 **	−0.298 **	0.105	−0.255 **
PSES	Self-efficacy		1	0.397 **	0.288 **	0.288 **	0.223 **	−0.107	−0.013	−0.325 **	−0.031	0.296 **	0.035	−0.092	0.092	−0.324 **
Empathy Scale for Teachers	Total%			1	0.966 **	0.056	0.696 **	−0.398 **	0.175 *	−0.535 **	−0.588 **	−0.046	−0.226 **	−0.083	0.191 **	−0.121
	CE%				1	−0.095	0.658 **	−0.452 **	0.184 **	−0.551 **	−0.662 **	−0.194 **	−0.281 **	−0.104	0.177 *	−0.037
	PAE%					1	−0.397 **	0.446 **	0.208 **	0.045	0.666 **	−0.095	0.573 **	−0.212 **	−0.084	−0.034
	NAE%						1	−0.658 **	0.033	−0.382 **	−0.821 **	0.362 **	−0.750 **	−0.079	−0.04	−0.262 **
Self-control scale	Total%							1	0.249 **	0.725 **	0.803 **	−0.318 **	0.634 **	0.314 **	−0.134	0.629 **
	IC%								1	0.228 **	0.171 *	−0.597 **	0.141 *	−0.477 **	−0.657 **	0.439 **
	GS%									1	0.423 **	−0.237 **	0.143 *	0.156 *	−0.448 **	0.787 **
	DC%										1	−0.141 *	0.705 **	0.012	−0.162 *	0.182 **
	SM%											1	−0.340 **	0.106	0.230 **	−0.679 **
	TI%												1	0.153 *	0.338 **	0.114
	ER%													1	0.563 **	0.113
	AU%														1	−0.401 **
	DM%															1

CE = cognitive empathy, PAE = positive affective empathy, NAE = negative affective empathy, IC = impulse control, GS = goal setting, DC = distraction control, SM = self-monitoring, TI = task initiation, ER = emotion regulation, AU = automaticity, DM = decision-making. * = *p* < 0.05, ** = *p* < 0.01.
